# Titanium uptake and incorporation into silica nanostructures by the diatom *Pinnularia* sp. (Bacillariophyceae)

**DOI:** 10.1007/s10811-014-0373-8

**Published:** 2014-07-24

**Authors:** Matilde Skogen Chauton, Lotte M. B. Skolem, Lasse Mork Olsen, Per Erik Vullum, John Walmsley, Olav Vadstein

**Affiliations:** 1 Department of Biotechnology, Norwegian University of Science and Technology (NTNU), SemSelands veg 6/8, 7491 Trondheim, Norway; 2 SINTEF Materials and Chemistry, Postboks 4760 Sluppen, 7465 Trondheim, Norway

**Keywords:** Silica, Titanium, Diatom, Bioincorporation, Frustulem, Nanostrucuture

## Abstract

Diatoms are an ecologically successful group within the phytoplankton, and their special feature is a biofabricated silica cell encasement called a frustule. These frustules attract interest in material technology, and one potential application is to use them in solar cell technology. The silica frustule with its nanoscaled pattern is interesting per se, but the utility is enhanced if we succeed in incorporating other elements. Titanium is an interesting element because its oxide is a semi-conductor with a high band gap. However, doping with relevant elements through bioincorporation is challenging, and it is necessary to understand the biology involved in element uptake and incorporation. Here we present data on bioincorporation of Ti into the silica frustules of the pennate diatom *Pinnularia* sp. (Ehrenberg) and show that the distribution of the incorporated Ti is inhomogeneous both between and within valves. More than a tenfold increase of Ti in newly synthesised valves was achieved, and increased Ti around the pores was confirmed by both EDS and EELS analyses. HAADF STEM spectroscopy revealed a grainy surface with amorphous silica particles of 4 to 5 nm in size. These observations are explained by what is known from the physico-chemical processes involved in biosilification and frustule formation, looking into it from a biological point of view.

## Introduction

Diatoms possess a cell encasement called the frustule, made of amorphous (i.e. non-crystalline) silica. The frustule consists of two valves, ornamented with species-specific patterns of pores and ribs on meso- and nanoscale dimensions. When the diatom cell divides each new cell will retain one old valve and synthesise one new valve. The actual role of the frustule remains a topic for discussion, but possible functions are light transmission, nutrient transport, gas exchange and grazer or virus protection. Amorphous silica is in principle transparent to visible light (which the living diatom use for photochemical energy synthesis), and it is suggested that the pore pattern provides a photonic crystal function that results in light focussing and transport into the chloroplasts (Yamanaka et al. 2008; De Stefano et al. 2009; Sclafani et al. 2013).

When diatoms grow exponentially (i.e. resource-unlimited growth), silicic acid (SiOH_4_) is actively transported via silica transporters (SITs) into the cytoplasm and then via transport vesicles into silica deposition vesicles (SDVs) (Annenkov et al. 2013). This process involves active transport against a concentration gradient from cytosol into the SDV and a pH gradient because the inside of the SDV has a lower pH. In the SDV, silicon (Si) polymerizes to silica (SiO_2_), and small building blocks are created. Exactly how these polymers are combined to form a frustule remains unknown (Del Amo and Brzezinski 1999; Zurzolo and Bowler 2001). Frustule construction is most probably a combination of genetically expressed Si transporter proteins that are involved in the transport of Si into the cell (Thamatrakoln and Hildebrand 2007) and post-translationally modified peptides (silaffins, silacidins) and long-chained polyamines involved in Si polymerization and construction of the frustule (Mock et al. 2008; Spinde et al. 2011); Kinrade et al. (2002) observed a transient organosilicon complex in the diatom *Navicula pelliculosa*: hexavalent Si was coordinated to nitrogen, but it was not possible to determine the localization of these complexes or if they were involved in frustule synthesis.

The highly modifiable frustule surface properties has been exploited in drug delivery technology (Aw et al. 2012), and the mesoporous frustule material has been applied as photocatalyst templates (Mao et al. 2014; He et al. 2013). The use of biogenic Si from diatoms in solar cell technology has been suggested, and the idea is extended to use the intact frustule itself and take advantage of the nanoscaled patterns of pores and rib structures (Jeffryes et al. 2011; Toster et al. 2013). The concentration of Si in aquatic environments is many times higher than other comparable elements, and the frustule composition likely reflects the availability of Si and also other elements: for example, the concentration of anions such as phosphorus is quite high in the frustules possibly because it plays a role in the formation of Si nanoparticles during frustule synthesis (Hildebrand 2008). From a material point of view, on the other hand, there are other elements such as in the semi-conductor TiO_2_ that would enhance the functionality of the frustule.

Increased concentrations of functional elements can be achieved by metabolic doping in living cells or by post-modifications such as thermal annealing. TiO_2_ (Jeffryes et al. 2008; Lang et al. 2013) and GeO_2_ (Gale et al. 2011) have been incorporated by bio-uptake or deposited on the frustules post-cleaning. GeO_2_ has been shown to inhibit diatom growth (Shea and Chopin 2007) and to interfere with frustule synthesis (Basharina et al. 2012). The uptake of other elements may be regulated as a function of concentrations, and there will be biological limits to the amount of a specific element that can be incorporated. Ti is not considered an essential element to organisms, but it has been discussed whether this is due to the fact that it is readily available, so we fail to detect its essentiality, or if it is truly non-essential. It is, however, bioactive (Buettner and Valentine 2012), and another consideration when trying to understand biogenic incorporation of various elements into the silica frustule is the potential harmful effects of, for example, TiO_2_ nanoparticles. The semi-conductive properties of TiO_2_ nanoparticles is desired in solar cells, but in living cells they are toxic in combination with UV light because the nanoparticles can lead to the formation of ROS (Miller et al. 2012). However, microalgae have a naturally high antioxidant protection because of their photosynthetic activity and concurrent oxygen formation. With the emerging field of nanomaterials, it has been shown that nanoparticles can accumulate in organelles such as the mitochondria and disrupt the electron transport chain with increased production of superoxide radicals (Li et al. 2003). It is therefore natural to assume that the uptake and transport of Ti into live cells is a controlled process in some way, where the Ti is directed to cell compartments where it is not damaging, e.g. the SDVs. No specialized Ti uptake or deposition proteins are known for diatoms, but mineralization of TiO_2_ by recombinant silaffins or silicateins has been demonstrated (Sumerel et al. 2003; Cole et al. 2006; Kroger et al. 2006).

Here we present data on uptake and incorporation of Ti in frustules of *Pinnularia* sp. (Ehrenberg), and we show that the photophysiology of *Pinnularia* cells was not adversely affected by the experimental setup and that Ti concentration is strongly enhanced in certain parts of the newly formed valves. Our work is a follow-up of Jeffryes et al. (2008) as we address some points raised in their discussion, in addition to providing new data on SiO_2_ particle size and the variability of Ti measurements. Our observations are interpreted in light of what is known about element uptake and deposition in living cells, and we discuss how this knowledge can be used to better understand both the biological function of a frustule and how to modify the frustule as a functional material.

## Materials and methods

### Cultivation, photophysiology and biomass

*Pinnularia* sp. (UTEX B679) was grown in filtered, autoclaved seawater amended with nutrients according to Guillard’s f/2-recipe for all elements except Si, which was reduced to 50 %. The natural seawater used here contains around 4 μM Si. Light was set at 130 μmol photons m^−2^ s^−1^ and 16:8 light/dark cycles. A smaller culture was kept for more than ten generations with regular dilutions to allow acclimatization before Ti uptake, and incorporation experiments were performed on larger experimental cultures. The cultivation setup consisted of borosilicate culture vessels of cylindrical shape with conical ends, with a diameter of 8 cm and a height of 50 cm (approximately 1,500 mL working volume). The volume of the experimental cultures was 1.1–1.2 L. Silicone stoppers and silicone hoses were used to ventilate the cultures, which were placed in a growth cabinet at 20 °C. Pressurized air (provided by aquarium pumps) was used to mix the cultures, using a gentle stream of bubbles from the bottom of the culture vessels. Macronutrients NO_3_, PO_4_ and Si were measured at selected time points during the acclimatization period to verify surplus (or in the case of Si, limitation before the experiments were initiated) using Merck Spectroquant test kits and SpectroquantPharo 100 spectrophotometer.

Instantaneous chlorophyll fluorescence (*F*_t_) and quantum yield (*Q*_y_) was monitored using a dual-mode AquaPen AP 100 (Photon Systems Instruments flurometer) to assess the biomass changes and photophysiological condition of the cells during cultivation and experiments. *Pinnularia* sp. has a tendency to cluster, and cell counts can be biased. Therefore, the consumed Si and the *F*_t_ measurements were used to assess changes in the biomass.

### Ti uptake and incorporation experiments

Ti uptake experiments were initiated when the Si concentration in the medium was < 0.5 μmol L^−1^ for two consecutive days and the cell numbers did not increase any further. Si limitation is known to induce cell cycle arrest in diatoms (Brzezinski et al. 1990), and the populations were expected to be highly synchronized at the start of the experiment. Visual inspection of samples at the start of the experiment showed some size differences, with cells of mainly three different sizes and with a mean cell size of 35.2 μm (CV 8.2 %, *n* = 67). A large number of paired cells were observed, in accordance with the Si limitation and cell cycle arrest before the final cell separation. The experiment was performed two times. Pure crystalline Ti(OH)_4_ was made according to Jeffryes et al. (2008), and a stock solution was prepared by dissolving the Ti(OH)_4_ in concentrated HCl (37 wt.%) with continuous stirring on a hot plate (80 °C). The stock solution was diluted with heated (80 °C) deionised water to a final concentration of 500 mM HCl/0.36 mM Ti. A solution of Na_2_SiO_3_ · 9H_2_O in 500 mM NaOH was made to balance the acidic Ti solution (final concentration 6.2 mM Si), and the two solutions were added in equal volumes to the experimental cultures using two Cole-Parmer 94900 Syringe pumps. The total Si and Ti solution volume was added drop-wise over 10 h, and the pH was between 8.0 and 8.4 during the experiments.

Control experiments were performed with (a) only medium (without cells) and addition of approximately 3.8 μmol L^−1^ Ti and 160 μmol L^−1^ Si to verify that both Si and Ti are recovered with the medium and do not adsorb to the glassware and (b) a culture of *Pinnularia* sp. grown in plastic bottles to determine the background of Ti in frustules without the borosilicate glassware as a possible source of Ti.

After centrifuging to remove the culture medium, organic material was removed from the biosilica using SDS/EDTA (50 mg L^−1^ in 100 mM EDTA solution), and the frustules were stored in methanol at −20 °C until analysed. One of the frustule samples from the Ti uptake experiments was chosen for determination of Ti by ICP-MS and analytical transmission electron microscopy (TEM). Control samples without addition of Ti were also analysed.

### Measurements of Ti by ICP-MS

High-resolution inductively coupled plasma mass spectrometry (HR ICP-MS; ELEMENT 2 instrument from Thermo Electronics) was used to determine the concentrations of Ti in seawater (background), cultivation medium and frustule samples, with and without incorporated Ti. A total of 2 mL of seawater or medium was filtered through 0.2-μm syringe filters into 15-ml centrifuge tubes (metal-free, VWR collection) and conserved with HNO_3_ (1 g, ultrapure). Samples were diluted 10× with deionised water before ICP-MS analysis. The reported measurements are the average of three technical replicates, with coefficient of variation (CV) of the mean (in %). In the medium samples, the CV was <5 % in all measurements.

Biosilica samples were rinsed with HNO_3_, transferred to PF containers and dissolved in concentrated HF acid (0.18 mL, grade supra pure). The dissolved frustules were diluted with 0.1 M HNO_3_ before ICP-MS analysis. The reported value is the average of two technical replicates, with CV 1.05 %.

The background value of Ti in *Pinnularia* frustules was determined from a culture grown in plastic bottles (to avoid any contribution from the glass material itself) but otherwise treated in the same way: The medium was the same as in the Ti uptake experiment (but no Ti was added), and the culture was aerated with pressurized air to promote mixing and gas transport. The background of Ti in seawater was determined from a sample of water collected from 80-m depth in the Trondheim fiord (the same water was used in the experiments).

### Analyses of Si and Ti by TEM

Transmission electron microscopy characterization was performed with two different instruments: (1) JEOL 2010 F operated at 200 kV and equipped with an 80-mm^2^ silicon drift detector (SDD) for X-ray energy dispersive spectroscopy (EDS) and (2) double C_s_ corrected cold field emission gun JEOL JEM-ARM200F operated at 200 kV. This instrument is equipped with a large solid angle Centurio SDD and a Quantum GIF with a dual electron energy loss (EEL) spectrometer. This setup allows for simultaneous acquisition of a high-angle annular dark field (HAADF) scanning transmission electron microscopy (STEM) image in combination with energy dispersive spectroscopy (EDS) and two different EEL regions. Hence, in a spectrum image, an EDS spectrum and two different EEL spectra are acquired and quantified in every pixel of the image.

The TEM samples were prepared by dispersing the frustules on a holey, amorphous carbon-coated Cu TEM grid. A selection of 21 frustules from the first experiment was analysed (aiming at either a valve or the girdle band zone) by EDS in the JEOL 2010 F to determine the average Ti concentration. Three frustules with high average Ti concentration were then selected from the second experiment for further analysis using the JEM-ARM200F to map and quantify the Ti concentration with high spatial resolution. Each measurement is the average Ti concentration across a region with a diameter of 1 to 2 μm (determined by the size/diameter of the electron beam) on the frustule, pointed at either valve or girdle band zones.

### Analysis of Si and Ti by X-ray photoelectron spectroscopy

Samples were also analysed by X-ray photoelectron spectroscopy (XPS) in order to determine the chemical state of the Ti at the surface and within the bulk of the frustules. A small drop of frustules in methanol was deposited on Al foil and dried. The analysis was performed using an Axis Ultra^DLD^ XP spectrometer (Kratos Analytical) and monochromatic Al Kα radiation. Pass energy of 160 eV was used for survey scans and 40-eV high-resolution scans of the individual core levels. Charge neutralization was applied, and the binding energy scale was referenced to the C 1 s component at 285 eV. The analysed area was approximately 300 × 700 μm. Ion beam sputtering was used to remove surface materials and reveal the bulk composition for analysis. Sputtering was performed using 2-kV Ar ions, and the analysis area after sputtering was approximately 100 μm in diameter.

## Results

### Growth and physiological status of *Pinnularia* sp

Under the environmental conditions provided here, the *Pinnularia* sp. cells maintained a specific growth rate of approximately 0.7 day^−1^, which corresponds to a doubling in cell numbers per day. Cell numbers counted at the start of the experiment was 4.11 × 10^4^ cells mL^−1^ (S.E. ±0.02, *n* = 2). An average of 1.13 pmol Si cell^−1^ was estimated from several measurements during the experiment (±0.07, CV = 5.87 %). A major fraction of the added Si was consumed during the first 24 h of the experiment or shortly after, corresponding to a biomass yield of approximately 4.20 − 10^4^ new cells mL^−1^, i.e. a doubling in biomass within the experimental period. *F*_t_ measurements showed a doubling also in fluorescence and corroborated a twofold increase in biomass during the experiment. *Q*_Y_ is a measurement of the efficiency of conversion of absorbed light into photochemistry, and it is used to assess the photophysiological status of the cells. *Q*_Y_ was 0.42 at the beginning of the experiment, indicating that the cells were in a reasonably healthy photophysiological state despite the Si deprivation previous to the experimental starting time (data not shown). The *Q*_Y_ then decreased to 0.30 during the 10 h of Ti/Si addition and thereafter increased to 0.37 where it remained stable for 48 h. When there was less than 1 μmol Si L^−1^ in the medium, the *Q*_Y_ sank to 0.25, indicating that the photophysiological condition was challenged.

### Dissolved Si and Ti in the cultures

**Fig. 1:**
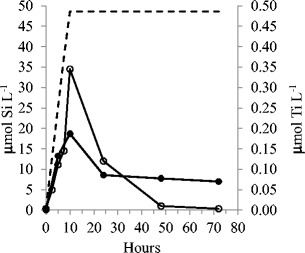
Dissolved Si (μmol L^−1^, *open circles*) and dissolved Ti (μmol L^−1^, *filled circles*) in the culture. The *dotted line* shows the addition of Si and Ti (calculated, not measured), which were added drop-wise for the first 10 h

A total of 48.6 ± 0.3 (SE, *n* = 2) μmol Si L^−^ of culture was added drop-wise to the experimental cultures during the first 10 h of the experiment, and 75 % of the added Si was removed from the medium by the cells during the first 24 h (Fig. 1). After 48 h, the dissolved Si (dSi) was low (1 μM), the biomass had ceased to increase and the experiment was terminated after 72 h. Simultaneously to the Si addition, 2.84 ± 0.01 μmol Ti L^−1^ (SE, *n* = 2) was added drop-wise to the experimental cultures. After 24 h, the medium contained only 0.086 μmol Ti L^−1^ (i.e. 97 % was taken up; Fig. 1). The background of Ti in the seawater used was 0.0064 μM (±0.0001 SE, *n* = 3) but somewhat higher in f/2 medium due to impurities in chemicals used to amend the seawater: 0.0094 μM (±0.0001 SE, *n* = 3). A control experiment without algae enriched with approximately 3.8 μmol L^−1^ Ti and 160 μmol L^−1^ Si showed that both Si and Ti stayed in the solution after 24 and 48 h. The concentrations measured were somewhat lower than the added dose, but not more than 10 % and within the errors in analysis and dosage of Si and Ti added.

### Si and Ti in frustules

**Fig. 2:**
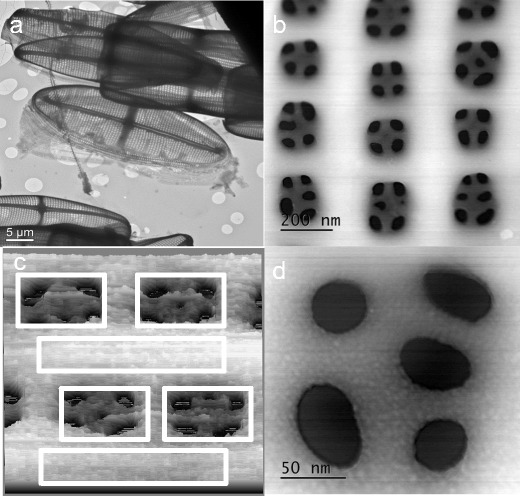
**a** Low-resolution, bright-field TEM image of cleaned *Pinnularia* frustules from one of the Ti uptake experiments. **b** Section of a *Pinnularia* sp. valve with pores. **c** High annular dark field (HAADF) scanning transmission electron spectroscopy (STEM) image of an area which includes four pore openings and surrounding SiO_2_. The *boxes* indicate where EDS/EELS measurements were performed, and in the four pore areas the average Ti was 1.06 (±0.101) at.%. In the two areas between the pores, the Ti was 0.21 (±0.050) at.%. **d** High annular dark field (HAADF) scanning transmission electron spectroscopy (STEM) image of one of the pores showing the granular appearance of the amorphous SiO_2_

The ICP-MS analysis showed that a sample of frustules grown in plastic flasks with f/2 medium and no Ti added contained 0.001 μM Ti (CV 17.7 %), which is comparable to the concentration of Ti observed in f/2 medium. A doubling of the biomass and concomitant removal of 2.76 μmol Ti L^−1^ of culture within the first 24 h of the experiment indicated a cellular content of 33.2 fmol Ti per cell in the new cells or 2.37 wt.% Ti/SiO_2_ (average over all frustules, not considering the difference between old and new valves, see the following related discussion). A low-resolution, bright-field TEM image (Fig. 2a) and a medium-resolution high-angle annular dark field (HAADF) scanning TEM (STEM) image (Fig. 2b) show the cleaned frustules with characteristic patterns of ribs and pores. The high-resolution HAADF STEM image of an area of pores revealed the granular surface of amorphous SiO_2_, with particles of 4 to 5 nm in diameter (Fig. 2d).

**Fig. 3:**
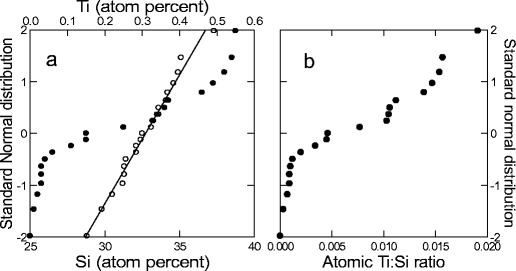
**a** Quantile plot based on the assumption of normal distribution of elements in 21 cleaned valves from *Pinnularia* sp. The linear fit confirms normal distribution of Si (*open circles*). The distribution of Ti is bi- or tri-phasic and confirms the different quantities of Ti in old vs. new valves (*filled circles*). The distribution was tested using Shapiro–Wilks test for normality, and *p* = 0.994 and 0.009 for Si and Ti, respectively. **b** Ti/Si ratios (atomic) in the 21 cleaned valves

EDS analysis of 21 randomly selected frustules gave an average atomic O/Si ratio of 2.04 ± 0.19 (SD), not significantly different from 2 (*p* = 0.357). The EDS analysis showed Si contents of 29 to 37 at.%, with an average of 33 at.% (±2 SD). A quantile plot suggested good fit (assuming a normal distribution) for the Si content (linear relationship; Fig. 3a). Ti content was from 0 to 0.55 at.%, with an average of 0.23 at.% (±0.20 SD) over all the frustules. Based on a quantile plot, the Ti content showed a bi- or tri-phased relationship consistent with a model of old valves with low Ti content and new valves (synthesised during the experiment) with high Ti content (Fig. 3a). The results also showed that there was an enrichment of Ti by a factor of 13 due to Ti addition: average Ti content of old valves was 0.023 ± 0.017 at.%, compared to 0.300 ± 0.148 at.% in new valves. The atomic ratios of Ti/Si in the 21 measurements were from 0 to 0.019 (Fig. 3b), and the distributions of Si and Ti were confirmed by Shapiro–Wilk test for normality with *p* = 0.994 and *p* = 0.009 for Si and Ti, respectively.

**Fig. 4:**
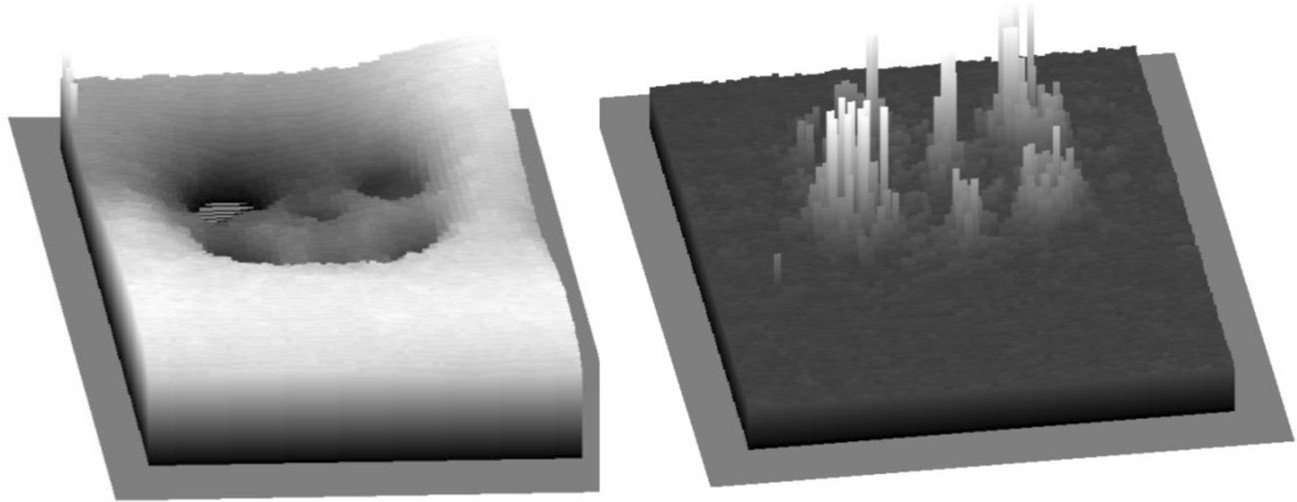
EDS thickness map (*left*) used to correct the EELS Ti map for thickness variations over the valve and pores; the resulting 3D image (*right*) shows the increased amounts of Ti localized around the pore openings in the *Pinnularia* sp. valve

**Fig. 5:**
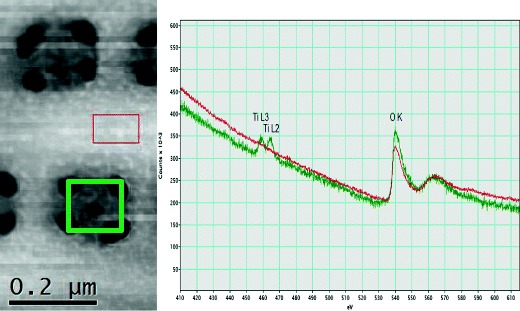
EELS data from pore region (*green square* and *line*) and between pores (*red square* and *line*) showing the O K peak around 540 eV present in both regions, while Ti L2 and Ti L3 (455–465 eV) are only observed in the spectra from the pore region

In order to provide finer detail about Ti distribution, three frustules with relatively high Ti concentrations were selected for high-spatial-resolution elemental mapping by simultaneous EDS and EELS. The EDS element maps from the three frustules showed significant differences between the Ti concentration in the pore linings and in the thicker SiO_2_ regions between the pores (Fig. 2c). The Ti concentration in the thin pore lining regions was >1 at.%, i.e. five times more Ti than in the thicker regions between the pores (Table 1). EELS measurements confirmed the inhomogeneous distribution of Ti in the biosilica with 0.00 at.% Ti in the bulk SiO_2_ (between pore areas) and 1.57 at.% Ti in the pore area/linings. The difference was evident also after correction for the variations in frustule thickness over the analysis area (Fig. 4). The energy loss spectra show the O K top around 540 eV present in both the pore region and the region between pores, while the Ti L2 and L3 tops (around 455–465 eV) are only present in the spectrum from the pore region (Fig. 5).

**Table 1:** Oxygen, silicon and titanium (at.%) and ratio of Ti to Si measured by EDS in either pores or rib area on cleaned valves of *Pinnularia* sp. The values are average of measurements (with STD in parenthesis) from three frustules that showed high Ti content

	O	Si	Ti	Ti/Si	Number, *n*
Pores	67.8 (2.30)	31.2 (2.29)	1.06 (0.101)	0.034	10
Ribs	68.0 (1.62)	31.8 (1.56)	0.21 (0.050)	0.007	4

**Fig. 6:**
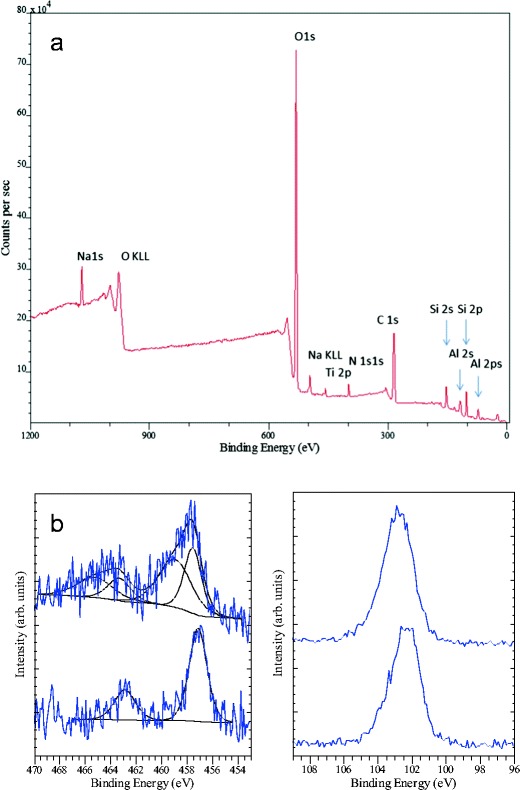
**a** XPS analysis of cleaned *Pinnularia* sp. frustules on an Al foil showing the overall surface composition of the frustules after drying. **b** The effect of Ar ion sputtering on the Ti 2p (*left panel*) and Si 2p (*right panel*) peaks; the initial analysis is shown in the *lower curve*, and the *upper curve* shows the analysis of the same area after Ar ion sputtering. The peaks appear in the same energy regions as in **a** but with the signal acquired from a smaller area for more detail

A XPS survey spectrum showed that, besides Si and O, C, N, Na and Ti were present as peaks that are associated with the surface of the frustules (Fig. 6a). The presence of Al and a proportion of the O signal are associated with the Al support foil. High-energy resolution scans from the Ti 2p peak region (Fig. 6b) before and after sputtering suggested the presence of Ti_2_O_3_ or TiO in the valve (Ti 2p1/2 ~ 463 and Ti 2p3/2 ~ 457 eV); after sputtering, the Ti peaks were consistent with the presence of TiO_2_ (Ti 2p1/2 ~ 464 and Ti 2p3/2 ~ 458 eV). The Si peak is consistent with the expected SiO_2_ composition, Si 2p ~103 eV.

## Discussion

Bioincorporation of Si in combination with other elements such as Ti, Ge or Zn is considered as a means to modulate the material properties of diatom frustules for use in solar cell technology. However, processes of element uptake and biosilicification must be understood in more detail before we are able to control the processes. Ti concentrations in the oceans are in the picomolar range, and natural marine phytoplankton contains up to 0.078 at.% Ti in the silica fraction (Martin and Knauer 1973). This is comparable to the lower amounts of Ti measured in the valves in our study (0.01–0.06 at.%), presumably the old valves that were produced prior to the Ti addition. The TEM images from this experiment show normally developed frustules without any obvious aberrations in the frustule shape or pore pattern, and thus growth in Ti-supplemented medium did not seem to interfere with the normal cell cycle and frustule synthesis. This was also supported by the measurements of fluorescence and quantum yield, which showed a doubling of the biomass and satisfactory photosynthetic performance during the first 24 h of the uptake experiment. From our measurements, the removal of Ti from the medium seemed to proceed with a rate proportional to that of Si, but how Ti is taken up or how fast it is taken up by the cells remains unknown. Based on the chemical similarities of Si(OH)_4_ and Ti(OH)_4_, it is reasonable to assume that Ti is taken up via the same transport system as Si, but not necessarily in a constant or foreseeable ratio. Our study of Ti uptake and bio-incorporation in *Pinnularia* sp. confirm that Ti is incorporated together with Si in the new frustules, in accordance with Jeffryes et al. (2008).

From our data, we see that there is a higher concentration of Ti around the pores on the valves (1.06 at.%), which is similar to the amounts and distribution patterns observed by Jeffryes et al. (2008). Our measurements include a correction for the uneven frustule thickness (Fig. 4) which Jeffryes et al. (2008) lacked, and the inhomogeneous distribution of Ti is therefore confirmed by our data. The fact that Ti is inhomogeneously distributed and concentrated around pores can be of use, for example, to enhance photon transport via the nano-scaled pores. If we understand why this happens, it would increase the possibility of biogenic manufacturing to produce frustules with special properties. Jeffryes et al. (2008) speculated that more Ti was deposited in the final stages of silica deposition due to Si pool exhaustion and slower rates of condensation of Ti to TiO_2_. In our experiment, the first generation of cells produced with Ti enrichment were not Si-limited and the Si pools therefore presumably non-exhausted (Fig. 1). Ti and Si were available in the culture medium in an atomic ratio of 0.10 in the beginning of the experiment, and when the addition of Ti and Si stopped after 10 h, the ratio changed to 0.06. Using the removed Ti and Si to estimate the intracellular pools, it seems that the ratio was high and stable (0.17–0.19) during the 10 h of Ti and Si addition. This time frame is coincident with finalization of the frustule and cell separation into two individual daughter cells in the cells that were synchronized due to Si depletion and the beginning of frustule synthesis in the next generation of cells (and concomitantly active Si uptake). The ratio sank to 0.06–0.08 from 24 h, and the next generation would then have a lower intracellular pool at the time when they finalize the fine structures of the frustules. Another explanatory model that is not related to concentrations of Ti and Si during the frustule synthesis period is that the uneven distribution of Ti in the frustules may be caused by the chemical quality of micelles or moulding structures involved in silica deposition and pore construction during frustule synthesis. Synthesis of the new valves in raphid pennate diatoms such as *Pinnularia* sp. starts from the central raphe and then proceeds outwards with ribs and crossing connections that form pores (Sato et al. 2011; Cox et al. 2012), using the cytoskeleton actin microfilaments to direct the deposition. Silica is deposited in micro-fibrils radiating out from a mid-line and bi-directionally towards the valve surfaces, and the raphe opening and the areolar pores are lined afterwards with a thick layer of silica that is more resistant to alkaline etching (Crawford et al. 2009). The processes that lead to the differentiation between the micro-fibrillar silica construction and the thick lining around openings have not been revealed, but increased concentration of Ti in these lining areas may be connected to these processes. Also, fluidic phase-separation theories and mathematical modelling have been used to show how the frustule architecture and pore pattern can arise by physio-chemical processes involving, e.g., polyamine micelles (Sumper 2002; Lenoci and Camp 2008; Willis et al. 2013), yet another explanatory model suggests that pores are constructed from molecular scaffolds or moulding structures onto which the Si is deposited or the scaffold creates an obstacle so that the Si deposition is interrupted at certain places, thus creating the pore patterns (Tesson and Hildebrand 2013). The exact composition of the scaffolds or moulding structures is not known, but chitin is found in close association with centric diatom valves and may play a role as a moulding agent (Brunner et al. 2009; Durkin et al. 2009).

The HAADF STEM image of a pore detail (Fig. 2d) revealed surface structures or particles of approximately 4 to 5 nm, which is very interesting considering that this is considerably smaller than the reported sizes of SiO_2_ precipitates made with natural diatom proteins. If the polymerization of Si occurs without salts or other ions present, colloidal particles of 2 to 3 nm in size are formed, and further development is dependent on pH and temperature (Coradin and Lopez 2003). Ions such as P, however, are known to influence the particle size of Si precipitates in vitro (Sumper et al. 2003), and Si particle size in frustules of living cells is also related to the different polyamines and proteins involved in Si precipitation (Kroger et al. 1999). A study by Lechner and Becker (2014) showed that different synthetic peptides resembling silaffins produced Si spheres of different sizes and also of different surface graininess depending on the numbers and positioning of lysine residues on the peptide. It is possible that we observed a grainy surface of larger Si polymers in the frustule surface. Also, Vertegel et al. (2004) demonstrated that the protein lysozyme adsorbed to Si nanoparticles of different sizes, and increasing particle size (40 or 100 nm) led to a higher degree of unfolding in the protein and concomitant decrease in activity. On small Si particles of 4 nm in diameter, however, the lysozyme did not unfold, and the activity was higher. The relation between the proteins involved in biosilification and particle sizes is not fully understood, but a similar mechanism with small particles and enhanced protein activity could play a role also in frustule synthesis.

EDS analysis was used to analyse the amounts of Ti in the biosilica, and the distribution of SiO_2_ showed a good fit with the assumption of normal distribution. Ti, on the other hand, was distributed in a bi- or even tri-phasic pattern (Fig. 3), and this observation is related to the occurrence of valves from different times in the culture period. Some valves are old (mother valves from before Ti addition), and other valves are newly synthesised “daughters” with Ti incorporated in the biosilica. The quantification of TiO_2_ around pores was considered approximate in the work of Jeffryes et al. (2008) due to scattering of the X-ray beam in the sample. In our measurements, higher spatial resolution was achieved by the application of EELS, and both EDS and EELS showed similar trends qualitatively with increased Ti around the pore linings and less Ti in the solid areas of biosilica between pores. The EELS measurements, however, indicated that the difference in Ti concentration from the pore regions to the regions between the pores is even larger than those quantified by EDS. This is because the EDS analysis suffered from stray radiation due to backscattered and diffracted electrons that hit other parts of the sample than the region illuminated by the beam. EELS measurements, on the other hand, do not suffer from such stray signals.

The XPS analysis of valves from the Ti incorporation experiment verified the presence of TiO_2_ in the bulk material of the valve before and after sputtering, and quantification of the peak areas gave a Ti/Si atomic ratio of ~0.05 (at). These results are consistent with the data from Lang et al. (2013) and Chrcanovic et al. (2012). High-energy resolution scans showed that the Si peak is consistent with the expected SiO_2_ composition, also after sputtering. A component of the Ti 2p peak appears at higher binding energy, which is indicative of a higher oxidation state such as Ti_2_O_3_/Ti^3+^ (Fig. 6b). There was a slight shift of 0.5–1 eV compared to the peaks measured before sputtering, but we cannot say with certainty from our data that the valve surface contains Ti_2_O_3_ or TiO. These results are difficult to interpret unambiguously due to charge compensation effects and the influence of the ion beam on the surface chemistry. The analysis is consistent with the presence of Ti^3+^ in the frustules, allowing for some uncertainty due to damage caused by the ion beam. The energy shift between oxidised and metallic Ti, however, is ~5 eV, and allowing for the uncertainty in energy reference, it is regarded as large enough to eliminate the possibility of a metallic component in the spectra. Mayer et al. (1995) described the influence of ion damage on the XPS spectrum obtained from TiO_2_ thin films. There are few studies of the biochemistry of Ti in cells or frustules (Buettner and Valentine 2012), but different Ti oxidation states in the frustule surface layers may be a result of the biochemical processes leading to the incorporation of Ti in the SiO_2_ or post-incorporation chemical modifications due to, e.g., cleaning with SDS, sample storage or exposure to air during the analyses. The presence of other elements such as C, N or Na can be related to remnants of organic molecules that were involved in the frustule synthesis (e.g. N-rich polyamines) and therefore tightly integrated into the SiO_2_. The SDS cleaning used here is considered a mild cleaning process that does not remove completely all the organic components of the valve. It is also possible that the organic remnants stem from other sources, and for example, adsorption of hydrocarbon or carbon oxygen species from the air onto the Ti surface of dental implants is known (Chrcanovic et al. 2012).

Our data confirm the uptake and incorporation of Ti into diatom nanostructures, and a relatively high doping effect is achieved in the pore lining areas. The inhomogeneous distribution of the element influences the application of Ti-doped frustules in, e.g., solar cell technology, and how this effect can be exploited needs more investigations into the material technological properties.
